# Association between high–risk fertility behaviors and neonatal mortality in Ethiopia: A multilevel mixed-effects logit models from 2019 Ethiopian mini demographic and health survey

**DOI:** 10.1016/j.puhip.2024.100515

**Published:** 2024-05-22

**Authors:** Hassen Ali Hamza, Abbas Ahmed Mohammed, Sadat Mohammed, Mohammed Feyisso Shaka

**Affiliations:** a Quality Improvement Unit Coordinator at Mekane-Selam General Hospital, Mekane-Selam, Ethiopia; b Department of Midwifery, College of Medicine and Health Sciences, Dilla University, Dilla, Ethiopia; c Department of Public Health, College of Medicine and Health Sciences, Debre Birhan University, Debre Birhan, Ethiopia; d School of Public Health, College of Medicine and Health, Madda Walabu University, Shashamane, Ethiopia

**Keywords:** Neonatal mortality, Multilevel mixed-effects logit model, High-risk fertility behaviors, Ethiopian mini-demographic and health survey 2019

## Abstract

**Objectives:**

This study aimed to explore the association between high–risk fertility behaviors and neonatal mortality in Ethiopia.

**Study design:**

A community-based cross-sectional study was conducted using data from the 2019 Ethiopian Mini-Demographic and Health Survey.

**Methods:**

Mixed-effects logit regression models were fitted to 5527 children nested within 305 clusters. The definition of high-risk fertility behavior was adopted from the 2019 EMDHS. The fixed effects (the association between the outcome variable and the explanatory variables) were expressed as adjusted odds ratios (ORs) with 95 % confidence intervals and measures of variation explained by intra-class correlation coefficients, median odds ratio, and proportional change invariance.

**Results:**

The presence of births with any multiple high-risk fertility behaviors was associated with a 70 % higher risk of neonatal mortality (AOR = 1.7, (95 % CI: 1.2, 2.3) than those with no high-risk fertility behavior. From the combined risks of high-risk fertility behaviors, the combination of preceding birth interval <24 months and birth order four or higher had an 80 % increased risk of neonatal mortality (AOR = 1.8, (95 % CI, 1.2, 2.7) as compared to those who did not have either of the two. The 3-way risks (combination of preceding birth interval <24 months, birth order 4+, and mother's age at birth 34+) were associated with approximately four times increased odds of neonatal mortality (AOR (95 % CI:3.9 (2.1, 7.4)].

**Conclusions:**

High-risk fertility behavior is a critical predictor of neonatal mortality in Ethiopia, with three-way high-risk fertility behaviors increasing the risk of neonatal mortality fourfold. In addition, antenatal follow-up was the only non-high fertility behavioral factor significantly associated with the risk of neonatal mortality in Ethiopia.

## 1 Introduction

WHO defines neonatal mortality as death within 28 days of birth [1]. It is a key indicator of children's health and wellbeing [2] and reflects a nation's socioeconomic status and healthcare service availability and accessibility [3,4]. Neonatal deaths reflect inadequate maternal and child health care, including inadequate professional service and a lack of standardized treatment for illnesses and complications after birth or in early childhood [5,6]. Most neonatal mortality causes are preventable with proper maternal and child health services [7].

Child mortality has decreased globally, but newborns still have unequal survival chances. Sub-Saharan Africa has 41 % of the global burden, and Southern Asia has 37 %, with an 80 % share of global neonatal mortality [[8], [9], [10]]. By 2030, SDG targets aim to reduce neonatal deaths to 12 per 1000 live births worldwide. Ethiopia is far from achieving this goal, with neonatal mortality still high [[11], [12], [13]]. 44 % of childhood deaths in Ethiopia occur in the first 28 days of life [14], making Ethiopia one of the five countries contributing to half of neonatal mortality globally [15].

High-risk fertility behavior (HRFB) is linked to neonatal mortality [16]. HRFB includes early or late childbirth, short inter-pregnancy intervals, and multiple births [16,17]. Reports show that HRFB causes many neonatal deaths [[18], [19], [20]]. Research in Ethiopia found neonatal mortality linked to short birth intervals and multiple pregnancies [21,22].

Numerous studies have been conducted in Ethiopia to explore the determinants of neonatal mortality and identified antenatal care, birth weight, hand washing, birth interval, birth order, maternal complications, breastfeeding characteristics, toilet facilities, and other individual and community-level factors [23,24,25,26,27,28,29]. However, most of these studies were conducted at the local level with a small number of participants and used standard logistic regression, which is methodologically questionable due to poor power, particularly when considering factors at different levels hierarchically. Limited studies have been conducted using multilevel analysis using previous Ethiopian demographic and health survey data [30,31,32], and these studies also failed to consider high-risk fertility behavior as a factor besides its possible significant effect on neonatal mortality and used older data sources.

## 2 Methods

### 2.1 Data source

This study used data from the 2019 Ethiopia Mini Demographic and Health Survey (EMDHS), the second EMDHS and fifth DHS in Ethiopia. The DHS program has conducted over 400 surveys in 90+ countries [33]. The 2019 EMDHS had 8885 women aged 15–49, with a 98.6 % response rate. The 2019 EMDHS children's recode dataset was accessed from https://www.dhsprogram.com/data/available-datasets.cfm.

### 2.2 Study area and period

Ethiopia, in Africa's Horn, is the continent's second most populous country with about 101 million people in 2020, ranking 12th globally. If the 2.6 % growth rate continues, the population will hit 122.3 million by 2030. Ethiopia is divided into 11 regions [34]. A national survey was conducted from March to June 2019.

### 2.3 Study design and sampling design

The Ethiopian Mini Demographic and Health Survey (EMDHS) 2019 employed a stratified two-stage cluster design. Clusters were sampled in the first stage and households in the second stage. Sample weights should be used to account for complex survey design, survey non-response, and post-stratification for representativeness of the samples [12]. Sample weights were used to make sample data representative of the population. The weight variable was v005, as the units of analysis were children. In Stata, the sample weight was calculated as wgt = v005/1000000.

**Fig. 1:**
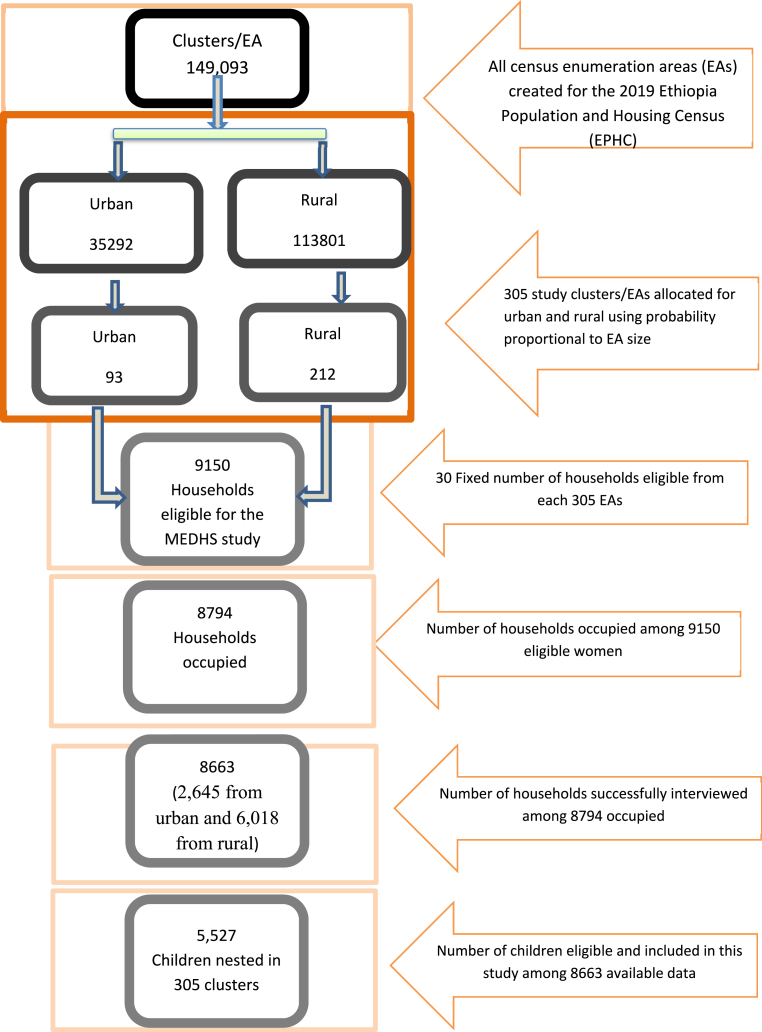
Diagrammatic presentation of the sampling procedure.

For EMDHS 2019 data, 8663 households (2645 urban, 6018 rural) were interviewed (99 % response rate). 8885 eligible women aged 15–49 were surveyed (99 % response rate). 5527 children in 305 clusters were included in the analysis (Fig. 1).

### 2.4 Measures of variables

#### 2.4.1 Dependent variable (outcome variable)

The outcome variable for this study was neonatal mortality, which refers to deaths at ages 0–29 days, including deaths reported at age zero months.

#### 2.4.2 Exposure

This study focused on maternal high-risk fertility behaviors. According to the 2019 EMDHS, these behaviors were classified as no extra risk, unavoidable risk, single high risk, and multiple high risk. Single high-risk fertility behaviors included mother's age <18, >34, birth interval <24 months, and birth order >3. Multiple high-risk fertility behaviors were combinations of two or more risk parameters [12].

#### 2.4.3 Control variables (potential confounders)

To estimate the effects of high-risk fertility behaviors on neonatal mortality, individual-level variables (proximate determinants: maternal factors, neonatal factors, and health system factors, and socioeconomic determinants: wealth index) and community-level variables (place of residence, contextual regions, distance from health facilities) that are theoretically and empirically linked to neonatal mortality were adjusted in our analysis.

### 2.5 Data management and analysis

#### 2.5.1 Descriptive analysis

Data were analyzed using Stata/SE version 14.0. Approximate-level weights (level-1 and level-2 weights) were applied to adjust the non-proportional allocation of the sample and non-response rate in all analyses. We used the -svy command to account for complex survey design (cluster sampling, stratification, and sampling weights). Categorization was performed for continuous variables, and re-categorization was performed for categorical variables. Descriptive analyses were performed to present frequencies and percentages.

#### 2.5.2 Multilevel logistic regression modeling

Based on the structure of the data (5527 women nested within 305 clusters/PSUs) and binary outcomes, a mixed-effects logistic regression modeling approach was fitted. Classical regression for nested data leads to statistical and conceptual problems (ecological and atomistic fallacies) [[35], [36]]. Thus, multilevel modeling is appropriate for nested data (hierarchical data).

Accordingly, mixed-effect models have both fixed effects (regression coefficients) and random effects (variance components). For this study, multilevel mixed-effects logit models were fitted using the svy-meqrlogit command in Stata 14/SE. Logit, like melogit, fits mixed-effects models for binary responses.

### 2.6 Parameter estimations

**Fixed effects** (regression coefficients): Measures of the association between the dependent and predictor variables. The results of fixed effects were expressed as adjusted odds ratios (AOR) with 95 % Confidence Intervals (CIs).

**Random effects (measures of variations):** The **measures of variation were expressed as** intraclass correlation coefficients (ICC) or Variance Partition Coefficient (VPC), Median Odds Ratio (MOR), and Proportional Change in Variance (PCV). ICC (VPC) is the proportion of cluster-level variance compared to the total variance [37].

### 2.7 Model selection (model Checking)

Information criteria (fit criteria) are used in model selection, such as DIC and AIC. AIC is calculated as −2*ln(likelihood) + 2 × k, where k is the number of estimated parameters. The model with the lowest AIC is the best fit, and deviance is −2 × ln (likelihood) [38].

### 2.8 Regression diagnostics methods

Variance inflation factors (VIF) were estimated to assess the risk of multicollinearity among predictor variables [35]. The VIF has a usual cutoff of 10. A VIF greater than 10 indicates the presence of multicollinearity among the predictors in the regression model.

## 3 Result

### 3.1 Socio-demographic and high-risk fertility behavior-related characteristics of the participants

A total of 5527 children nested within 305 primary sampling units (clusters) from 21 strata were included in this analysis. Of these, 2842 (51.42 %) were male. From our sample, 2132 (38.57 %) fell into birth with any single high-risk category, and 1178 (21.3 %) fell into births with any multiple-risk category (Table 1) (see Table 2).

**Table 1:** Distribution of children born in the 5 years preceding the survey by HRFB risk category and proximate factors, Ethiopia Mini-DHS 2019.

Variables	Frequency (%)
Sex of child	
Male	2842 (51.42 %)
Female	2685(48.58 %)
HRFB Risk Category	
No extra risk	1275 (23.1 %)
unavoidable first birth risk	942 (17.0 %)
any single high-risk category	2132 (38.57 %)
any multiple risk category	1178 (21.3 %)
Maternal educational level	
Primary education or less	4918 (89 %)
Secondary education and above	609 (11 %)
Place of residence	
Urban	1367 (24.7 %)
Rural	4160 (75.3 %)
Attended 4+ ANC visits	
No	3839 (69.4 %)
Yes	1688 (30.6 %)

ANC: Antenatal care.

**Table 2:** Any single high-risk fertility behaviors and neonatal mortality using MEDHS 2019.

Variables	COR [95 % CI]	Full Model: AOR [95 % CI]
HRFB: any single high-risk fertility behaviors		
Mother's age at birth		
≥18 years	1	1
<18 years	1.9 (1.2, 2.9)	1.8 (1.1, 2.8)
Mother's age at birth		
≤ 34 years	1	1
>34 years	1.5(1.1, 2.3)	1.9 (1.2, 2.9)
Birth spacing (preceding birth interval)		
≥24 months	1	1
<24 months	1.7 (1.3, 2.4)	1.8 (1.3, 2.5)
Birth order		
<4	1	1
≥ 4	0.9 (0.7,1.2)	0.8 (0.5, 1.1)
Individual and community-level factors		
Sex of child		
Male	1	1
Female	0.8 (0.6, 1.06)	0.8 (0.6, 1.04)
4^+^ ANC visits		
No	1	1
Yes	0.5 (0.4, 0.7)	0.5 (0.3, 0.8)
Place of delivery		
Health facility	1	1
Home	1.1 (0.8, 1.4)	0.8 (0.5, 1.05)
Place of residence		
urban	1	1
rural	1.1 (0.7, 1.6)	0.9 (0.6, 1.3)
Maternal education level		
Primary & below	1	1
Secondary^+^	0.5 (0.3,0.9)	0.6 (0.3, 1.04)

COR: Crude odds ratio; AOR: Adjusted odds ratio; CI: Confidence interval.

### 3.2 Measures of association (fixed effects) between high-risk fertility behaviors (HRFB) and neonatal mortality using 2019 EMDHS, obtained from multilevel logistic models

#### 3.2.1 Any single high-risk fertility behaviors and neonatal mortality

After adjusting for confounding factors, the link between high-risk fertility behaviors and neonatal mortality was evaluated. Infants born to mothers under 18 or over 34 had a higher mortality risk [AOR (95 % CI:1.8 (1.1, 2.8) and AOR (95 % CI:1.9 (1.2, 2.9)] respectively. Data shows short birth intervals (<24 months) significantly raised neonatal mortality risk [AOR (95 % CI:1.8 (1.3, 2.5)] while 4+ ANC visits reduced it [AOR (95 % CI:0.5 (0.3, 0.8)]. However, child's sex, delivery location, residence, and maternal education didn't significantly impact neonatal mortality.

#### 3.2.2 Combined risks of high-risk fertility behaviors (any multiple-risk category) and neonatal mortality

After adjusting for confounders, children with any multiple risk categories had a twofold higher risk of neonatal mortality [AOR (95 % CI:1.7 (1.2, 2.3)]. Children with spacing <24 months, order 4+, and age 34+ had a fourfold higher risk of neonatal mortality [AOR (95 % CI:3.9 (2.1, 7.4))], and those with spacing <24 months and order 4+ had an 80 % extra risk [AOR (95 % CI:1.8 (1.2, 2.7)]. Table 3 showed that two combinations (spacing <24 and age <18, and order 4+ and age 34+) had no significant effect on neonatal mortality. The highest risk of neonatal mortality was seen with 3-way risk (spacing <24, order 4+, and age 34+). (Table 3).

**Table 3:** High-risk fertility behaviors: specific combinations of risk factors (any multiple risk category) using MEDHS 2019.

Variables	COR [95 % CI]	Full Model: AOR [95 % CI]
**HRFB: any multiple-risk category**		
Births with any multiple-risk category		
No	1	1
Yes	1.8 (1.3, 2.4)	1.7 (1.2, 2.3)
Double risk, spacing <24 and age <18		
No	1	1
Yes	0.9 (0.2, 3.8)	0.9 (0.2, 3.9)
Double risk, birth order 4+ & age 34+		
No	1	1
Yes	1.1(0.7, 1.8)	1.3 (0.8, 2.0)
Double risk, spacing <24, order 4+		
No	1	1
Yes	1.73 (1.2, 2.6)	1.8 (1.2, 2.7)
3-way risk, spacing <24, order 4+, age 34+		
No	1	1
Yes	3.8 (2.02,7.05)	3.9 (2.1, 7.4)

#### 3.2.3 Random effects (measures of variations)

The intercept-only model (null model) showed 11.3 % of neonatal mortality variation was due to differences between clusters (VPC/ICC). The null model's median odds ratio (MOR) was 1.9. Clusters (primary sampling units) varied in neonatal mortality. Model 4 explained 4.8 % of the mortality differences within clusters, due to the combined effects of level-1 and level-2 predictors. (Table 4).

**Table 4:** Random effects (Measures of variations) for Neonatal Mortality at the Primary Sampling Unit (Cluster) Level by a mixed-effects logistic regression modeling, EMDHS 2019.

Random-effects Parameters	Null Model (intercept-only model)	Final Model (Model 4)
Cluster level variance (SE)	0.42 (0.15)	0.4(0.15)
PCV (%)	Reference	4.8 %
ICC or VPC (%)	11.3 %	10.8 %
MOR	1.9	1.8
Fit Criteria		
Loglikelihood	−887.69	−866.57
DIC	1775.4	1733.1
AIC	1779.4	1757.1

## 4 Discussion

This study aimed to identify predictors of neonatal mortality, with a primary focus on high-risk fertility behaviors. The analysis revealed that high-risk fertility behaviors were significant variables in explaining neonatal mortality in Ethiopia. Specifically, young women under 18 years and older women over 34 years were found to be nearly twice as likely to experience neonatal deaths. Previous studies conducted in Ethiopia have consistently highlighted young maternal age as a major risk factor for neonatal mortality [11,31,[39], [40], [41], [42]]. Proposed physiological pathways also suggest that adolescent mothers are more prone to delivering low birth weight and premature babies, both of which are significant contributors to neonatal mortality [38,43,44]. Additionally, young mothers may lack adequate biological or nutritional maturity, which further increases the risk of neonatal mortality. Moreover, young women in developing countries often exhibit insufficient child-rearing skills and face challenges in accessing healthcare for themselves and their children [45,46].

Furthermore, emerging evidence indicates that the later-life health outcomes of offspring born to mothers of advanced maternal age (34+ years) may be adversely affected. Studies have demonstrated that advanced maternal age is associated with cardiovascular maladaptation and an increased risk of adverse neonatal outcomes, including preterm birth, fetal growth restriction, and low birth weight. These factors collectively elevate the risk of neonatal mortality [47].

Short birth intervals (less than 24 months) were linked to an 80 % higher risk of neonatal mortality. This is consistent with previous studies showing that short birth intervals are the main risk factor for child mortality [40,[48], [49], [50]]. Short birth intervals may exacerbate the risk of neonatal mortality due to maternal depletion from successive pregnancies and lactation, as well as increased competition for household resources among closely spaced children [[51], [52], [53]].

On the other hand, antenatal care (ANC) follow-up identified as a critical factor in reducing the risk of neonatal mortality in this study. Women who attended four or more ANC visits had a 50 % lower risk of neonatal death, highlighting the importance of early detection and timely treatment of pregnancy complications. Studies in Ethiopia and other countries found similar results [31,[54], [55], [56]]. This likely reflects the ability to detect complications during pregnancy and timely treatment if women attend ANC visits [57,58].

The study also examined the cumulative effect of multiple high-risk fertility behaviors on neonatal mortality. Children born to mothers exhibiting three or more high-risk fertility behaviors faced a significantly higher risk of mortality. For neonates from mothers with spacing <24 months, birth order 4+, and maternal age 34+, the risk of mortality was fourfold higher. A Bangladesh DHS study found that multiple high-risk fertility behaviors (HRFBs) had major impacts on neonatal outcomes [59]. A multi-country study on DHS data in Asia and Africa (excluding Ethiopia) showed that each HRFB factor raised neonatal mortality risk, with no significant difference in odds ratio between single and combined risks [40]. This underscores the urgent need for targeted interventions aimed at addressing high-risk fertility behaviors to reduce neonatal mortality rates in Ethiopia.

## 5 Conclusion

This study utilized large-scale representative data from the Ethiopian Demographic and Health Survey to investigate the impact of high-risk fertility behavior on neonatal mortality. The findings underscore the significant contribution of high-risk fertility behavior to neonatal mortality in Ethiopia, highlighting the importance of addressing this issue through targeted interventions. Stakeholders and policymakers must collaborate with maternal health programs to design and implement effective strategies to mitigate the impact of high-risk fertility behaviors on neonatal mortality rates. However, it is essential to acknowledge the limitations of the study, including potential biases in the EDHS data, the cross-sectional nature of the analysis, and incomplete variables in the mini-EDHS dataset, which may have resulted in the omission of important confounders.

## Points for practice from the findings

Implementing targeted interventions to address high-risk fertility behaviors among young and older women to reduce neonatal mortality rates in Ethiopia.

Prioritizing early detection and management of pregnancy complications through increased antenatal care (ANC) follow-up, aiming for four or more ANC visits.

Promote optimal birth spacing of at least 24 months to mitigate the risk of neonatal mortality associated with short birth intervals.

## Availability of data and materials statement

The dataset generated and/or analyzed during the current study is accessed from DHS on reasonable request.

## Funding

No grant was received for this study from any funding agency.

## Authors’ contributions

**HAH:** carried out conceptualization of the study, requested permission to download the EMDHS 2019 dataset, performed formal analysis, developed the design of methodology, interpretation of the result, visualization of data, and wrote the final manuscript. **AAM:** writing the research proposal, carrying out the literature review, formal analysis, and methodology, and drafting the original manuscript. **SM:** carried out the literature review, formal analysis, methodology and reviewed the final manuscript. All authors read and approved the final manuscript. **MFS:** Carried out a literature review, reviewed and critics of the proposal, guided the statistical analysis, carried out an interpretation of the result and made intellectual inputs, involved in the write-up of the final research work and write-up of the manuscript.

## Consent for publication

Not applicable.

## Ethics approval

The data was accessed from EDHS Measure based on an official request with information about the planned work on the data. All methods were carried out following relevant guidelines and regulations.

## Declaration of competing interest

The authors declare that they have no competing interests. All authors agreed on the submission of the manuscript.

## List of abbreviations

**AIC**: Akaike's information criterion
**AOR**: Adjusted odds ratio
**CI**: Confidence interval
**COR**: Crude odds ratio
**DIC**: Deviance information criterion
**EA**: Enumeration areas
**EDHS**: Ethiopia Demographic and Health Survey
**EMDHS**: Ethiopia Mini-Demographic and Health Survey
**DHS**: Demographic and Health Survey
**HRFB**: High-Risk Fertility Behaviors
**ICC**: Intra-class correlation coefficient
**MOR**: Median odds ratio
**PCV**: Proportional change in variance
**PSU**: Primary sampling unit
**SE**: Standard error
**SDG**: Sustainable Development Goal
**VIF**: Variance inflation factor
**VPC**: Variance partition coefficient
**WHO**: World Health Organization
